# Fc-optimized CD276 antibody enhances NK cell activation against non-small cell lung cancer

**DOI:** 10.3389/fimmu.2025.1624751

**Published:** 2025-07-31

**Authors:** Sylwia A. Stefańczyk, Xenija Kaiser, Ilona Hagelstein, Samuel Holzmayer, Latifa Zekri, Susanne Jung, Melanie Märklin

**Affiliations:** ^1^ Clinical Collaboration Unit Translational Immunology, Department of Internal Medicine, University Hospital Tübingen, Tübingen, Germany; ^2^ Cluster of Excellence iFIT (EXC 2180) ‘Image-Guided and Functionally Instructed Tumor Therapies’, Eberhard Karls University Tübingen, Tübingen, Germany; ^3^ Department of Peptide-based Immunotherapy, Institute of Immunology, University of Tübingen and University Hospital Tübingen, Tübingen, Germany

**Keywords:** non-small cell lung cancer (NSCLC), CD276 (B7-H3), NK cell, ADCC, immunotherapy, Fc engineering, PD-1/PD-L1 non-responders, monoclonal antibody

## Abstract

Non-small cell lung cancer (NSCLC) is one of the most common and lethal cancers worldwide, with a poor prognosis for many patients, especially in advanced stages. The development of immune checkpoint inhibitors (ICIs) has transformed treatment strategies for NSCLC. ICIs targeting PD-1/PD-L1 have shown substantial bene!t, but these therapies are not effective in all patients and are also associated with significant side effects. One promising target for NSCLC immunotherapy is CD276 (B7-H3), an immune checkpoint molecule that is highly overexpressed in many tumors, but minimally expressed in healthy tissues. CD276 is involved in immune escape mechanisms, tumor growth, and metastasis, making it an attractive target for patients unresponsive to PD-1/PD- L1-directed therapies. To address the limitations of T cell-based ICIs, natural killer (NK) cells are being explored as a complementary strategy, as they directly lyse tumor cells through antibody-dependent cellular cytotoxicity (ADCC). Here, we present an Fc-optimized CD276 antibody, 8H8_SDIE, which enhances NK cell reactivity by improving its binding affinity to CD16. In our preclinical studies 8H8_SDIE specifically binds to CD276 on NSCLC cell lines, resulting in significant NK cell activation, characterized by increased expression of CD69 and CD107a, and the secretion of cytotoxic mediators such as IFNγ, perforin, and granzyme B. These findings suggest that 8H8_SDIE may provide a novel therapeutic option for patients with CD276-positive NSCLC, particularly those who have failed to respond to conventional T cell-activating ICIs. By engaging NK cells, this approach could overcome the limitations of PD-1/PD-L1-directed therapies, offering a new way to combat ICI-resistant tumors.

## Introduction

Lung cancer, of which non-small cell lung cancer (NSCLC) accounts for 85-88%, has both the highest incidence and mortality rate of all cancers worldwide, largely driven by the prevalence of smoking, its main causative agent (1). While the 5-year survival rate is around 64% in the localized stage, it drops to a mere 9% in the distantly metastasized stage, which, considering that around 53% of cases are primarily diagnosed with distant metastases, explains the dismal prognosis of this disease (2). In recent years, the treatment landscape for metastasized disease has been rapidly changing, with targeted therapies and immunotherapies gaining more and more ground in metastatic NSCLC (3–5).

Among these, immune checkpoint inhibitors (ICIs) have arguably the biggest part in revolutionizing the treatment of even early-stage NSCLC (6), with nivolumab, pembrolizumab, ipilimumab and atezolizumab all being used in different subentities at different stages. However, while certain subgroups show durable responses to these treatments, others show limited or no benefits (7, 8). Likewise, while ICIs are claimed to have lower toxicity rates than standard chemotherapy (9–11), a significant proportion of patients still suffer severe, sometimes even mortal, side effects (12, 13) that require specific guidelines for their management (14) and often compromise or even prevent further treatment. This underscores the need to develop new strategies for those patients hitherto excluded from the benefits of the established immunotherapeutic regimens.

In recent years we have successfully evaluated and validated several modified monoclonal antibodies (mAbs) with increased ability to induce antibody-dependent cellular cytotoxicity (ADCC) in various clinical entities (15–17). ADCC is a crucial mechanism through which mAbs exert their therapeutic effects, primarily mediated by natural killer (NK) cells (18, 19). Enhanced ADCC can be achieved by modifying the fragment crystallizable (Fc) region of mAbs. Two important strategies for this enhancement include optimization of glycosylation patterns and substitution of specific amino acids within the Fc region (e.g. S239D/I332E, SDIE) (20). A notable example of the former is the FDA-approved glycol-optimized CD20 mAb obinutuzumab, which is used to treat B cell malignancies. By increasing the affinity of the Fc region for Fcγ receptors (FcγRs), particularly the activating FcγRIIIa (also known as CD16a), the efficacy of ADCC can be improved. This enhanced binding has a greater effect on activating receptors compared to inhibitory receptors, such as FcγRIIb (CD32b) (21).

However, to broaden the application of these improved mAbs to different cancer types, it is also essential to identify specific tumor-associated antigens that are largely present on tumor cells but minimally expressed in healthy tissues. One such antigen of interest is CD276, also referred to as B7-H3. This immunological checkpoint molecule has shown tumor-restricted expression in several malignancies, including NSCLC (22, 23), leading to its recognition as a potential target for novel therapeutic interventions. CD276 expression is associated with poor prognosis, likely due to its role in inhibiting the activity of T cells and NK cells (24–26), thereby providing a rationale for targeting this antigen in therapeutic strategies aimed at enhancing anti-tumor immunity.

In this study, we analyze the expression levels of CD276 in NSCLC cell lines, while also validating a newly developed Fc-optimized CD276 monoclonal antibody, known as 8H8_SDIE, which enhances NK cell activity and cytotoxicity against NSCLC.

## Materials and methods

### Peripheral blood mononuclear cells and cell lines

Peripheral blood mononuclear cells (PBMC) were obtained from healthy volunteer donors. PBMC were isolated by Ficoll density gradient centrifugation (Thermo Fisher Scientific, Waltham, MA, USA). After isolation, PBMC were cryopreserved in liquid nitrogen and then randomly selected for each experimental run. Prior to use, cryopreserved PBMC were thawed and cultured in RPMI 1640 medium (Thermo Fisher Scientific) at 37°C in 5% CO_2_ for 24 hours to ensure viability for experiments. All participants gave written informed consent in accordance with the Declaration of Helsinki, and the study was approved by the Committee of the University of Tübingen.

The non-small cell lung cancer (NSCLC) cell lines (A549, NCI-H226, NCI-H460) were obtained from both the German Collection of Microorganisms and Cell Cultures (Braunschweig, Germany) and the American Type Culture Collection (Manassas, VA, USA). To maintain quality standards, mycoplasma contamination screening was performed every three months, and cell line authenticity was verified by flow cytometry-based immunophenotyping according to suppliers’ protocols.

### Production and purification of antibody

A CD276-specific monoclonal antibody (mAb) with the SDIE modification, 8H8_SDIE, and a corresponding iso-SDIE control were generated. An anti-CD276 mAb (clone 8H8) and a control mAb (clone MOPC21) were chimerized with the human immunoglobulin G1/K constant region. The mAbs were optimized for Fc function by introducing S239D/I332E modifications as described previously (27). The light and heavy chain plasmids for these mAbs were prepared using the EndoFree Plasmid Maxi kit (Qiagen, Hilden, Germany) according to the manufacturer’s guidelines. Antibody production was carried out in the ExpiCHO cell system (Gibco, Carlsbad, CA) in accordance with the recommended protocols.

Antibody purification from the culture supernatants was achieved by protein A affinity chromatography (GE Healthcare, Chicago, IL), followed by preparative size exclusion chromatography (HiLoad 16/60 Superdex 200, GE Healthcare). To confirm antibody purity and quality, analytical size exclusion chromatography (Superdex 200 Increase 10/300 GL, GE Healthcare) was performed alongside SDS-PAGE using 4–12% gradient gels (Invitrogen, Carlsbad, CA) and Bio-Rad’s Precision Plus protein standards (Hercules, CA).

### Flow cytometry analysis

Cells were first blocked with human or mouse IgG (Merck KGaA, Darmstadt, Germany), followed by incubation with mouse anti-human CD276-PE/Cy7 (clone MIH42, BioLegend, San Diego, CA, USA), 8H8_SDIE, or their respective isotype controls (BD Pharmingen, San Diego, CA, USA). Secondary labeling was performed with either goat anti-mouse PE (DAKO, Glostrup, Denmark) or goat anti-human PE (Jackson ImmunoResearch, West Grove, PA, USA). Natural killer (NK) cells were stained with fluorescence-labeled antibodies CD3-APC (clone SK7, BD Pharmingen) and CD56-PE/Cy7 (clone HCD56, BioLegend). For intracellular IFNγ and TNF detection, cells were cultured with GolgiStop and GolgiPlug (BD Biosciences, Heidelberg, Germany), followed by CD56 staining as described above, cell fixation and permeabilization with the Fixation/Permeabilization Solution Kit (BD Biosciences), and subsequent staining with IFNγ-BV421 (clone B27, BioLegend).

To assess target cell lysis, NSCLC cells were labeled with 2.5 mM CellTrace™ Violet proliferation dye (Thermo Fisher Scientific) prior to co-culture with PBMC from healthy donors, with or without the addition of antibodies (1 µg/mL each). Silicone beads (Merck KGaA) were used to standardize test volume measurements. Dead cells were excluded from the analysis by staining with 7-AAD (BioLegend) or LIVE/DEAD™ Fixable Aqua (Thermo Fisher Scientific). Flow cytometry data were acquired on either a FACS CANTO II or FACS Fortessa instrument (BD Biosciences) and analyzed using FlowJo_10 software (FlowJo LLC, Ashland, OR, USA). Specific fluorescence intensity (SFI) values were calculated by dividing the mean fluorescence intensity (MFI) of the antigen by the MFI of the isotype control, with surface positivity defined as SFI ≥ 1.5.

### Evaluation of NK cell activation, degranulation and cytokine secretion

To assess NK cell activation independently of tumor cells, a high binding plate (Greiner Bio-One, Frickenhausen, DE) was coated overnight with 10 µg/mL of 8H8_SDIE monoclonal antibody, with PBS added to the control wells. PBMC from eight healthy donors were then added and incubated for 24 hours. After incubation, the cells were stained with CD56 and CD3 to identify NK cells, CD107a as a degranulation marker, and fixable aqua dye to assess cell viability.

To evaluate target-dependent NK cell activation, degranulation, and cytokine release, PBMC from healthy donors were co-cultured with NSCLC cells at an effector-to-target (E:T) ratio of 2.5:1, using 200,000 NSCLC cells and 500,000 PBMC, with treatments applied at a concentration of 1 µg/mL. To assess NK cell degranulation, GolgiPlug and GolgiStop (BD Biosciences) were added to the co-culture, cells were collected after 24 hours, stained for CD107a-PE (clone H4A3, BD Pharmingen) and analyzed by flow cytometry. Activation markers CD69-PE (clone FN50, BD Pharmingen) and CD25-PE (clone BC96, BioLegend) were used to assess NK cell activation at 24 and 72 hours. NK cells within the PBMC population were identified by gating for the CD3^-^CD56^+^ subset.

To analyze cytokine secretion, supernatants from the 24-hour co-culture were collected and analyzed for levels of granzyme A, granzyme B, perforin, granulysin, TNF, IL-2, IFNγ, and IL-10 using the Legendplex assay (BioLegend), according to the manufacturer’s instructions.

To determine IFNγ secretion after 72 h, supernatans from co-cultures of PBMC from healthy donors with NSCLC cell lines (E: T of 2.5:1) were analyzed by ELISA.

Plates were coated overnight with a 0.3 µg/mL anti-human IFNγ mAb (Pierce Endogen®, Thermo Fisher Scientific) in carbonate-bicarbonate buffer (pH 9.6, Sigma-Aldrich, USA), blocked with 1% BSA-PBS (PAN Biotech, Aidenbach, DE), and washed. Supernatants were added in triplicate and plates were incubated for 2 hours at room temperature (RT). After washing, a 0.5 µg/mL secondary antibody (anti-human IFNγ mAb biotin-labeled, Pierce Endogen®, Thermo Fisher Scientific) in 1% BSA-PBS was added for 2 h, followed by poly-HRP-Streptavidin (1:80000, Research Diagnostics, Baileys Harbor, WI, USA) for detection. Plates were developed using TMB substrate (Medac, Wedel, Germany), and IFNγ was quantified using Spectra Max ID5 system (Molecular Devices, Silicon Valley, CA, USA). Concentrations represent the mean of triplicate measurements.

### Analysis of NK cell cytotoxicity

The lysis of NSCLC cells by PBMC from healthy donors, with or without 8H8_SDIE or MOPC_SDIE (1 µg/mL), was evaluated using the DELFIA Cell Cytotoxicity Assay (Perkin Elmer, Waltham, MA, USA) after a 2-hour incubation according to standard protocols (15). Specific lysis rates were calculated using the formula:

100 × (experimental release – spontaneous release)/(maximum release – spontaneous release). Lysis rates are presented as the mean of technical triplicates, along with the standard error of the mean, unless otherwise noted.

Long-term, real-time cytotoxicity was evaluated at 15 min intervals over 150 h using the xCELLigence RTCA system (Roche Applied Science, Penzberg, Germany). For this assay, NSCLC cells were seeded in 96-well plates for 24h prior to co-culture with PBMC from healthy donors at an effector-to-target (E:T) ratio of 40:1, with or without the specified monoclonal antibodies (1 µg/mL).

### Statistics

Unless otherwise stated, results are presented as the mean ± SEM of replicates or individual data points. Statistical significance was evaluated using methods such as Student’s t-test, one-way ANOVA, the Mann-Whitney test, or the log-rank test, as appropriate. All analyses were carried out using GraphPad Prism version 10.1.1, with significance thresholds set at p-values below 0.05 (*p < 0.05, **p < 0.01, ***p < 0.001). Statistical significance (p < 0.05) is indicated for groups with sufficient sample sizes (n ≥ 3), while non-significant comparisons are not marked.

## Results

### CD276 surface expression on NSCLC cell lines

**Figure 1:**
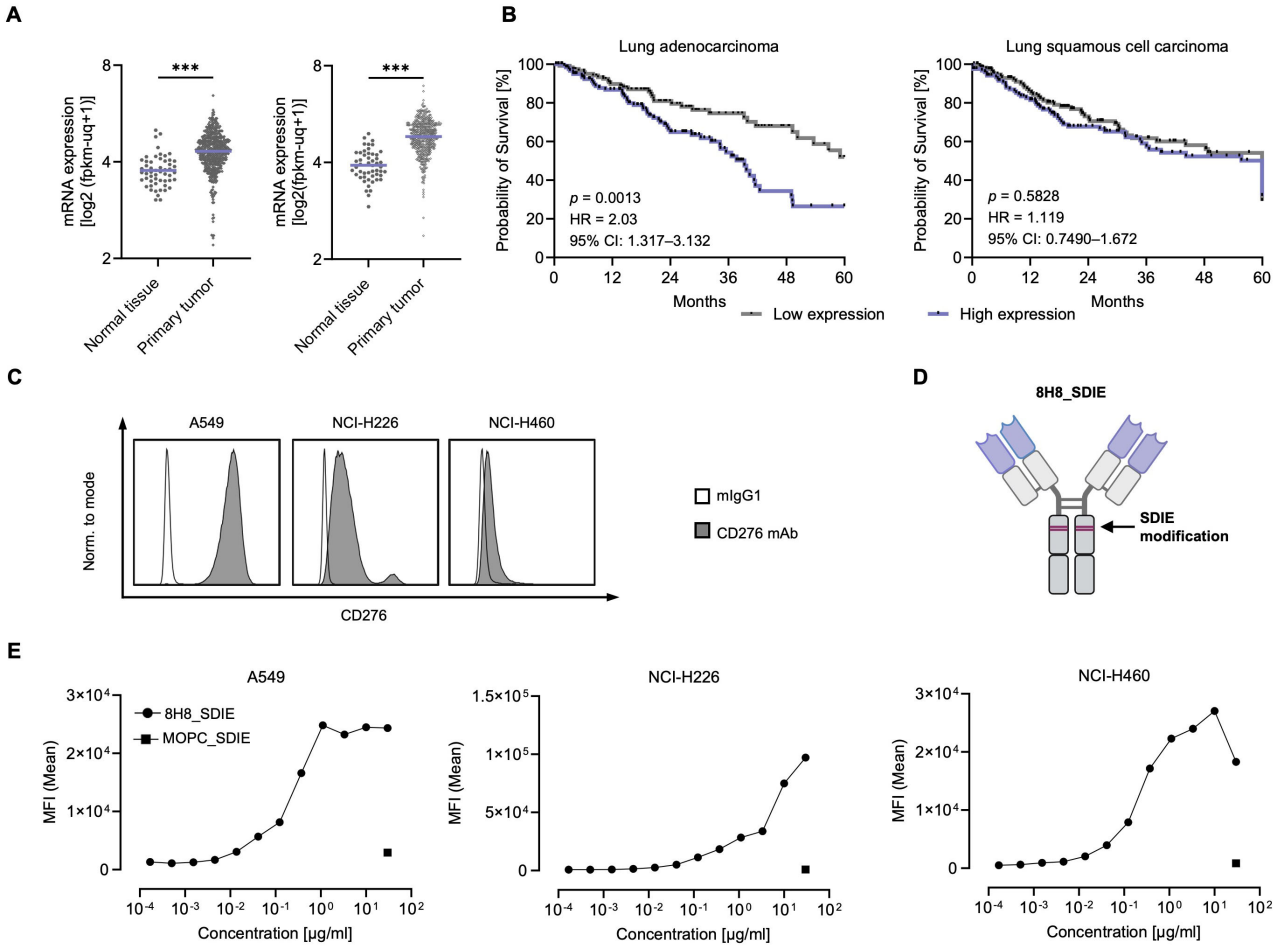
Characterization of CD276 expression in NSCLC cell lines. **(A)** mRNA expression data from the TCGA TARGET GTEx database were processed using the USCS Xena platform (70). The left panel shows CD276 mRNA expression in healthy lung tissue (n = 54) and primary lung adenocarcinoma tissue (n = 491) for. The right panel shows CD276 mRNA expression in healthy tissues (n = 51) and lung squamous cell carcinoma tissues (n = 501). **(B)** Kaplan-Meier survival curves for lung adenocarcinoma and lung squamous cell carcinoma based on CD276 expression. Survival data obtained from the USCS Xena platform (TCGA dataset). Five-year survival curves of lung adenocarcinoma patients (n = 251, left panel) and lung squamous cell carcinoma patients (n = 244, right panel) for the highest and lowest CD276 expression quartiles are shown. **(C)** Flow cytometric analysis of CD276 surface expression on the depicted NSCLC cell lines using commercially available CD276-Pe/Cy7 and a corresponding murine IgG1 isotype control is shown. Representative histograms from one out of three independent experiments with similar results are shown. **(D)** Schematic representation of the engineered anti-CD276 antibody with a modified Fc region designed for increased affinity to CD16 (8H8_SDIE). Created with BioRender.com. **(E)** Flow cytometric analysis of 8H8_SDIE mAb titration on selected NSCLC cell lines using MOPC_SDIE as isotype control. ***p < 0.001.

As a first step, we analyzed CD276 mRNA expression using TGCA TARGET GTEx data sets from tumor and matched normal tissues to assess relative expression levels. This analysis included data sets for 54/491 lung adenocarcinoma (normal/primary tumor) and 51/501 lung squamous cell carcinoma samples (**Figure 1A**). Compared to normal tissue, CD276 RNA expression was significantly increased in all tumors examined. Using survival data derived from UCSC Xena based on the TCGA datasets, we evaluated the impact of CD276 expression levels on overall survival (OS) in patients with lung adenocarcinoma and lung squamous cell carcinoma by Kaplan-Meier survival analysis (**Figure 1B**). Patients were stratified into high and low CD276 expression groups. For lung adenocarcinoma, survival analysis revealed a significant difference between high and low CD276 expression groups. Patients with high CD276 expression had a shorter median survival of 39.3 months, while the median survival for the low expression group was indeterminate, suggesting longer survival. Furthermore, high CD276 expression was associated with a twofold increased risk of death, confirming its potential prognostic role in lung adenocarcinoma. Similar trends but less pronounced without reaching statistical significance were observed in lung squamous cell carcinoma, where the survival curves for high and low CD276 expression levels were more closely aligned.

Next, we evaluated CD276 surface expression on various NSCLC cell lines, including A549, NCI-H226, and NCI-H460, by examining the specific binding of a commercially available CD276 antibody (**Figure 1C**). Flow cytometry analysis indicated that CD276 expression was high on A549 cells, moderate on NCI-H226 cells, and low on NCI-H460 cells. We then evaluated the binding of a humanized CD276 monoclonal antibody, clone 8H8, termed 8H8_SDIE, which included the S239D/I332E modification to enhance its affinity for the CD16 Fc receptor on NK cells (**Figure 1D**). As a control, we employed an Fc-optimized mAb with irrelevant target specificity, designated MOPC_SDIE. Binding of 8H8_SDIE to NSCLC cells was further evaluated by dose titration using the A549, NCI-H226 and NCI-H460 cell lines, with results showing that approximately 1 μg/mL of the antibody was sufficient to achieve maximal binding in all cases (**Figure 1E**).

### Induction of NK cell activation with 8H8_SDIE in NSCLC

**Figure 2:**
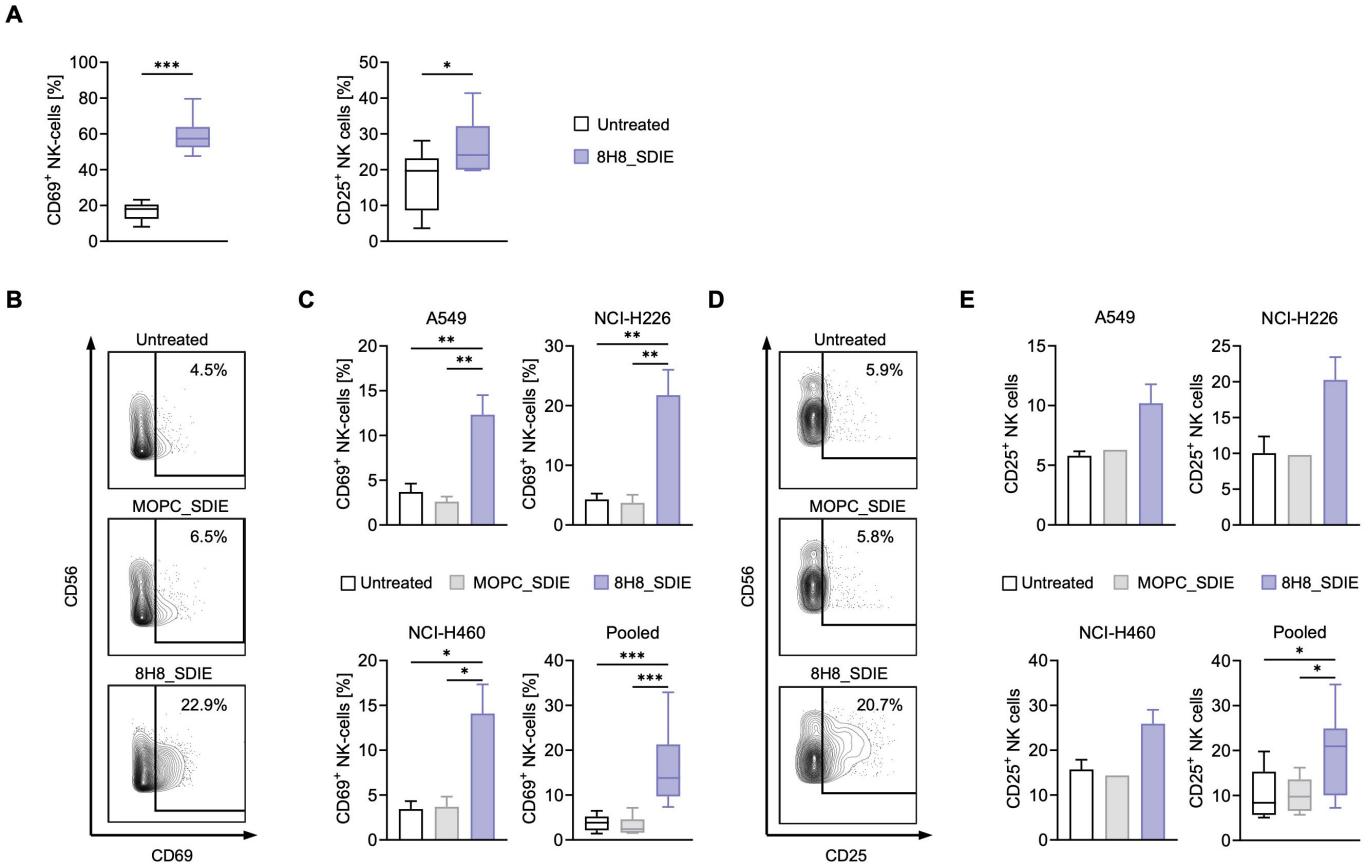
Induction of NK cell activation by Fc-optimized CD276 antibody against NSCLC cell lines. **(A)** 8H8_SDIE was immobilized on high binding plates and NK cell activation was assessed by flow cytometry by measuring the expression of CD69 and CD25 after incubating PBMC from healthy donors (n = 8) for 24 h. **(B-E)** PBMC from healthy donors (n = 4) were co-cultured with the indicated NSCLC cell lines (E:T 2.5:1) with or without 8H8_SDIE or MOPC_SDIE control (both at 1 µg/mL). **(B)** Representative flow cytometric results for CD69 expression of NK cells after co-culture with NCI-H226 cells. **(C)** NK cell activation was determined by CD69 expression after 24 h. Separate and pooled data of the indicated NSCLC cell lines incubated with PBMC from healthy donors. **(D)** Representative flow cytometric results for CD25 expression of NK cells after co-culture with NCI-H226 cells. **(E)** NK cell activation was analyzed by CD25 expression after 72 h. Individual and pooled data of the indicated NSCLC cell lines incubated with PBMC from healthy donors. *p < 0.05; **p < 0.01; ***p < 0.001.

To evaluate whether 8H8_SDIE could enhance NK cell activation independent of target cells, 8H8_SDIE mAb was immobilized and PBMC from healthy donors, containing NK cells as an effector population were added for 24 hours. After incubation, flow cytometric analysis of NK cells showed significantly increased CD69 (left panel) and CD25 (right panel) expression after 24 h of treatment with 8H8_SDIE compared to controls, indicating enhanced NK cell activation (**Figure 2A**).

Next, we investigated the ability of 8H8_SDIE to promote NK cell responses against NSCLC cells. PBMC from healthy donors were incubated with various NSCLC cell lines, in the presence of 8H8_SDIE or its isotype control, MOPC_SDIE. Flow cytometry results after 24 hours showed a significant increase in CD69 expression on NK cells treated with 8H8_SDIE compared to the MOPC_SDIE control, indicating increased activation against all NSCLC lines tested (**Figures 2B, C**). After 72 hours of incubation, treatment with 8H8_SDIE resulted in a significant increase in CD25 expression on NK cells, while no effect was observed with the isotype control on all tested NSCLC cell lines (**Figures 2D, E**).

### Stimulation of NK cell activity targeting CD276^+^ NSCLC cell lines

**Figure 3:**
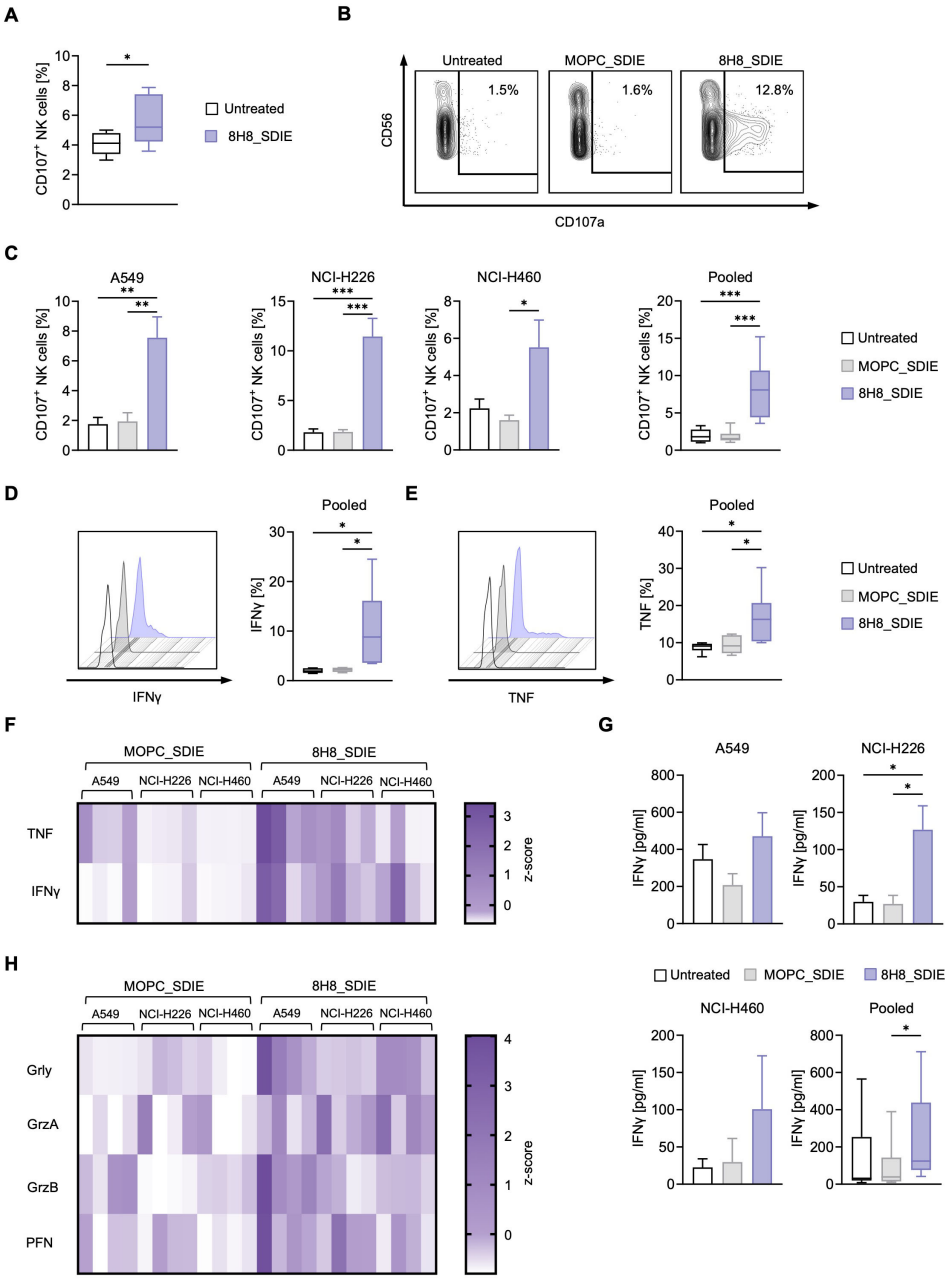
NK cell reactivity is induced by an Fc-optimized CD276 antibody against NSCLC cell lines. **(A)** 8H8_SDIE was immobilized on high binding plates and NK cell degranulation via CD107a was assessed by flow cytometric analysis after 4 hours using PBMC from healthy donors (n = 8). **(B-H)** PBMC from healthy donors (n = 4) were co-cultured with the indicated NSCLC cell lines (E:T 2.5:1) with or without 8H8_SDIE antibody or the MOPC_SDIE control (both at 1 µg/mL). **(B)** Representative flow cytometric results for CD107a expression of NK cells after co-culture with NCI-H460 cells at 4 h. **(C)** NK cell degranulation was analyzed by CD107a expression at 4 h. Individual and pooled data of the NSCLC cell lines with PBMC from healthy donors. **(D, E)** Intracellular IFNγ and TNF expression of NK cells (n = 4) after co-culture with NSCLC cell lines (E:T 2.5:1) was characterized after CD3^-^CD56^+^ counterstaining and analysis by flow cytometry after 4 h. **(D)** Exemplary histograms of NK cells after co-culture with A549 cells for IFNγ expression (left panel) and pooled data from different NSCLC cell lines for IFNγ expression (right panel). **(E)** Exemplary histograms of NK cells after co-culture with A549 cells are shown for TNF expression (left panel) and pooled data from different NSCLC cell lines for TNF expression (right panel). **(F, H)** Supernatants of the respective co-cultures were analyzed after 24 h for the release of **(F)** the immunoregulatory molecules IFNγ and TNF and **(H)** the effector molecules granzyme A (GrzA), granzyme B (GrzB), perforin (PFN) and granulysin (Grly) by Legendplex assay. The heat maps show individual values for the indicated NSCLC cell lines and different PBMC donors (n = 4). **(G)** IFNγ ELISA was performed with supernatants of PBMC from healthy donors (n=4) co-cultured with NSCLC cell lines (n=3) for 72 h at an E:T ratio of 2.5:1. *p < 0.05; **p < 0.01; ***p < 0.001.

Next, we evaluated whether 8H8_SDIE could enhance NK cell reactivity independently of target cells by adding PBMC from healthy donors to plates with immobilized 8H8_SDIE. Flow cytometric analysis after 4 h showed a significant increase in NK cell degranulation compared to controls, as indicated by CD107a expression (**Figure 3A**).

Target-specific NK cell degranulation was assessed by coculturing PBMC from healthy donors with NSCLC cell lines in the presence or absence of 8H8_SDIE and MOPC_SDIE. Flow cytometry showed robust CD107a induction with A549, NCI-H226, and NCI-H460 cell lines (**Figures 3B, C**). Intracellular flow cytometry analysis of the effector cytokines IFNγ and TNF, which have direct anti-tumor properties, showed a significant increase in IFNγ and TNF expression by NK cells upon treatment with 8H8_SDIE (**Figures 3D, E**). The increased levels of IFNγ and TNF were further confirmed by their increased release into the culture supernatants at 24 hours and 72 hours for (**Figures 3F, G**). In addition, treatment with 8H8_SDIE resulted in increased secretion of key cytotoxic molecules, including granulysin, granzyme A, granzyme B, and perforin compared to controls (**Figure 3H**).

### NK cell cytotoxicity against CD276^+^ NSCLC induced by 8H8_SDIE

**Figure 4:**
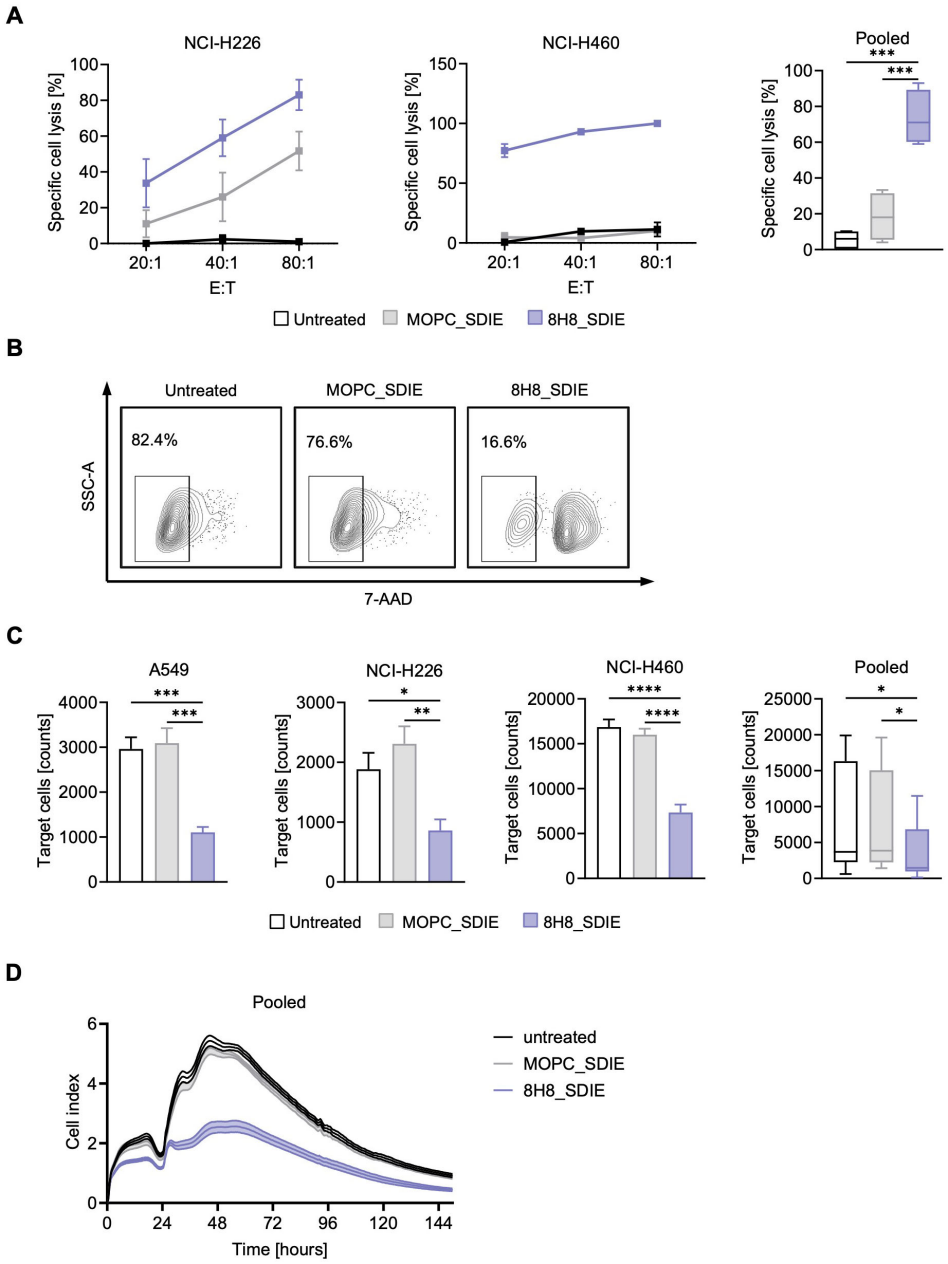
Induction of NK cell cytotoxicity by Fc-optimized CD276 antibody against primary NSCLC cells. PBMC from healthy donors were co-cultured with the indicated NSCLC cells with or without 8H8_SDIE mAb or MOPC_SDIE control (both 1 µg/mL). **(A)** Targeted lysis of NSCLC cells was quantified by Europium-based cytotoxicity assays after 2 h of incubation. The left and middle panels show example data for NCI-H226 and NCI-H460 cells and a PBMC donor at different E:T ratios. The right panel shows pooled data from A549, NCI-H226 and NCI-H460 cell lines with different PBMC donors (n = 4) (E:T 40:1). **(B, C)** Flow cytometry-based lysis of NSCLC cells was analyzed after 24 h by labeling of target cells and counterstaining of dead target cells. **(B)** Representative results for NCI-H226 cells after 24 h co-culture are shown by contour plots. **(C)** Lysis of NSCLC cell lines with different PBMC donors (n = 4) showing individual and pooled results. **(D)** NCI-H460 cells were cultured with PBMC from healthy donors (n = 4) (E:T 40:1) for 150 h and long-term killing of NSCLC cells was determined using a real-time cell analysis system (xCELLigence). *p < 0.05; **p < 0.01; ***p < 0.001; ****p < 0.0001.

We next investigated whether the enhanced NK cell reactivity induced by 8H8_SDIE leads to increased target cell lysis. PBMC from healthy donors were co-cultured with NSCLC cell lines in the presence or absence of 8H8_SDIE mAb. Europium-based cytotoxicity assays at 2 hours demonstrated a significant increase in target cell lysis with 8H8_SDIE treatment in all NSCLC cell lines tested (**Figure 4A**). Similarly, long-term 24-hour flow cytometry-based assays confirmed the ability of 8H8_SDIE to induce robust lysis of NSCLC cells (**Figures 4B, C**). Additionally, real-time cell imaging over a 150-hour period further validated the cytotoxic effects of 8H8_SDIE (**Figure 4D**). Notably, coculture experiments with CD276-negative HL-60 cells showed no activation, degranulation, or specific lysis induced by 8H8_SDIE compared to the isotype control (**Supplementary Figure S1**).

## Discussion

Immunotherapy has significantly transformed the treatment landscape for non-small cell lung cancer (NSCLC), offering new hope to patients with advanced or previously uncontrollable disease. Immune checkpoint inhibitors (ICIs) targeting PD-1 and PD-L1 have become a cornerstone of therapy, improving survival and quality of life in various molecular and clinical subgroups of NSCLC patients, regardless of PD-L1 expression levels (28, 29). Despite their widespread use, ICIs face substantial challenges, particularly with respect to primary resistance. Landmark trials have shown that a large proportion of patients fail to respond to initial ICI therapy, with primary resistance rates as high as 78% in PD-L1 positive patients (30–32). Secondary resistance is also a concern, as some patients develop adaptive resistance mechanisms over time (33). In addition to resistance, ICIs can trigger significant immune-related adverse events (irAEs) due to broad immune system overstimulation, leading to severe autoimmune toxicities, that can affect multiple organ systems and, in some cases, become life-threatening (34, 35). Given these barriers, there is an urgent need for therapeutic innovations that go beyond PD-L1-based approaches and more effectively address both primary and acquired resistance.

To overcome these challenges, novel immunotherapeutic targeting strategies such as anti-tumor antibodies are urgently needed. To address this issue, we have developed an Fc-optimized monoclonal antibody, 8H8_SDIE, targeting the tumor-associated antigen CD276. Optimized Fc receptor binding enhances antibody-dependent cellular cytotoxicity (ADCC) by increasing binding to CD16a on NK cells, while reducing interactions with the inhibitory FcγRIIb receptor. Our Fc-engineered monoclonal antibody, 8H8_SDIE, represents a significant advance in immunotherapy by incorporating the S239D/I332E (SDIE) modification to enhance ADCC, which has been demonstrated with multiple antigens in various cancers, including leukemia, colorectal cancer, breast cancer, and sarcoma (15, 27, 36–40). This is consistent with previous advances in Fc-optimized mAbs such as obinutuzumab (CD20; NCT02393157), FLYSYN (anti-FLT3; NCT02789254) (41), margetuximab (Her2; NCT01828021), MEN1112 (CD157; NCT02353143), and tafasitamab-cxix (CD19; NCT02399085), which have also demonstrated clinical efficacy.

CD276 is broadly expressed on tumor cells and with relatively limited expression in normal tissues, making it an ideal candidate for antibody-based therapy, particularly Fc-engineered antibodies designed to enhance NK cell-mediated tumor cytotoxicity (42, 43). CD276 was originally characterized as a costimulatory molecule, but recent evidence supports its predominant function in immune suppression immune evasion, metastasis and angiogenesis (44–46), solidifying its role as a multifaceted therapeutic target (43, 47). Notably, CD276 expression is frequently observed in NSCLC tumors lacking PD-L1, making it particularly relevant for patients who do not benefit from PD-1/PD-L1 inhibitors (48). Elevated CD276 levels correlate with worse overall survival, particularly in adenocarcinoma subtypes, highlighting its relevance in treatment-resistant tumors (45, 49).

Several CD276-targeting strategies have entered preclinical and clinical evaluation, including antibody-drug conjugates (ADCs) (e.g., MGC018: NCT037219596, DS7300a: NCT04145622), radiolabeled mAbs (e.g., ^131^I-8H9: NCT03275402, NCT04022213; ^177^Lu-DPTA Omburtamab: NCT04315246, NCT04167618), Fc-optimized mAbs (e.g., MGA271, Enoblituzumab: NCT02923180, NCT02475213, NCT04634825; DS-5573a: NCT02192567, clinical trial discontinued), CAR-T cells and bispecific antibodies (CC-3: NCT05999396, MGD009: NCT02628535) (50–56). These approaches have shown promising anti-tumor activity, but face different difficulties. For instance, Zhang et al. recently developed a CD276-directed antibody-drug conjugate (ADC) that exhibited anti-tumor activity in non-small cell lung cancer (NSCLC) models, primarily by delivering a cytotoxic MMAF payload (42). Although effective, this ADC strategy relies on toxin conjugation, which can lead to systemic toxicity and off-target effects. In contrast, our Fc-optimized antibody, 8H8_SDIE, induces potent NK cell-mediated cytotoxicity solely through immune effector engagement. This payload-free alternative may reduce adverse effects while preserving anti-tumor efficacy. In general, ADCs and radiolabeled mAbs can exhibit off-target toxicity due to their potential accumulation in non-target tissues, particularly during hepatic and renal clearance processes, which can lead to significant side effects and limit their therapeutic efficacy (57, 58). In addition, ADCs often have a narrow therapeutic window and can cause dose-limiting toxicities that are not necessarily related to the target antigen, complicating their clinical application (58, 59). CAR-T cell therapies, while promising, face challenges such as high production costs, complex manufacturing processes and serious side effects such as cytokine release syndrome and neurotoxicity, which may hinder their widespread use in the treatment of solid tumors (60–62).

In summary, these studies suggest that CD276-targeted therapies, are very promising and show robust antitumor activity with manageable toxicity profiles. Therefore, we reasoned that our Fc-optimized mAb 8H8_SDIE will be a promising drug candidate for further evaluation in NSCLC patients, especially those who do not express PD-L1 or are unresponsive to ICI therapy.

While the conducted *in vitro* and *ex vivo* studies using healthy donor NK cells provide valuable insights, they do not fully capture the complexity of the NSCLC tumor microenvironment, which is characterized by immune suppression (63, 64) and NK cell dysfunction (65, 66). Advanced *in vivo* models, such as patient-derived xenografts (PDX) or humanized mice, are essential to recapitulate human tumor dynamics and evaluate therapeutic efficacy under clinically relevant conditions. Our previous *in vivo* studies using 8H8_SDIE in AML xenograft models (17) demonstrated a favorable safety profile, confirming the absence of off-target immune activation or cytokine release while effectively inhibiting leukemia progression. These findings support the potential of 8H8_SDIE as a therapeutic agent also in NSCLC with a reduced risk of severe adverse events, such as cytokine release syndrome. However, further research, including combinatorial approaches with ICIs, is essential to evaluate the long-term impact of NK cell therapies, particularly with regard to tumor evolution and resistance mechanisms. In addition, comprehensive biodistribution studies are needed to further confirm the safety profile of 8H8_SDIE, particularly in tissues with low CD276. Another critical aspect is the improvement of NK cell persistence, which typically ranges from a few days to four months, which could be achieved by immunocytokines targeting IL-2 or IL-15 to enhance NK cell proliferation (67–69).

The potent and selective anti-tumor activity of our 8H8_SDIE in preclinical settings underscores its potential as a promising therapeutic option and could provide substantial benefit to patients with NSCLC and other CD276-positive malignancies, potentially overcoming the limitations of PD-L1 dependent strategies.

## Data Availability

The raw data supporting the conclusions of this article will be made available by the authors, without undue reservation.

## Supplementary material

Supplementary Figure 1 NK cell-mediated cytotoxicity against the CD276-negative HL-60 cell line after treatment with Fc-optimized 8H8_SDIE. **(A, B)** PBMCs from healthy donors (n = 3) were co-cultured with the CD276-negative HL-60 cell line at an E:T ratio of 2.5:1 for 24 h in the presence of 8H8_SDIE or the MOPC_SDIE isotype control (both at 1 μg/mL). **(A)** Representative flow cytometric data showing CD69 expression on NK cells. **(B)** Representative data for CD107a expression as a marker of NK cell degranulation. **(C)** Specific lysis of HL-60 cells was measured using an Europium-based cytotoxicity assay after two hours of coculture with PBMCs from healthy donors (n=3) at an effector-to-target ratio of 40:1. There was no significant difference in lysis between 8H8_SDIE and the SDIE isotype control, indicating CD276-dependent activity.
