# Supplementary figures and images for: Fc-optimized CD276 antibody enhances NK cell activation against non-small cell lung cancer
Source: Front Immunol. 2025 Jul 31;16:1624751. doi: 10.3389/fimmu.2025.1624751 (PMC12350344; doi:10.3389/fimmu.2025.1624751)

**A**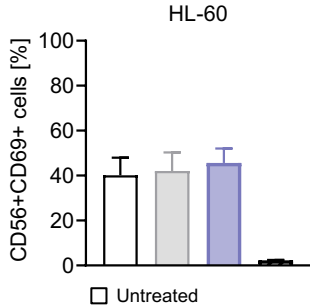**B**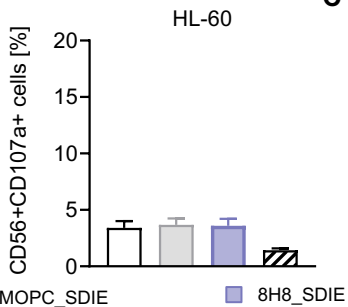**C**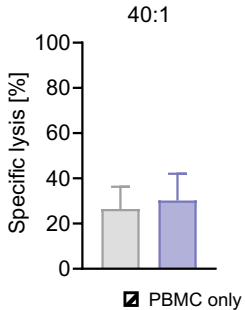

Supplement: Supplementary Figure 1 — NK cell-mediated cytotoxicity against the CD276-negative HL-60 cell line after treatment with Fc-optimized 8H8_SDIE. (A, B) PBMCs from healthy donors (n = 3) were co-cultured with the CD276-negative HL-60 cell line at an E:T ratio of 2.5:1 for 24 h in the presence of 8H8_SDIE or the MOPC_SDIE isotype control (both at 1 μg/mL). (A) Representative flow cytometric data showing CD69 expression on NK cells. (B) Representative data for CD107a expression as a marker of NK cell degranulation. (C) Specific lysis of HL-60 cells was measured using an Europium-based cytotoxicity assay after two hours of coculture with PBMCs from healthy donors (n=3) at an effector-to-target ratio of 40:1. There was no significant difference in lysis between 8H8_SDIE and the SDIE isotype control, indicating CD276-dependent activity. [file Image1.pdf]
